# Negative affectivity in university students and its relationship with academic performance and professional outlook after COVID-19

**DOI:** 10.1590/0034-7167-2024-0040

**Published:** 2024-08-30

**Authors:** Bruna de Oliveira Alves, Bárbara Guimarães Lourenço, Bianca Bacelar Assis Araújo, Luana Vieira Toledo, Rafael Lopes Chaves, Érika de Cássia Lopes Chaves, Tânia Couto Machado Chianca, Caroline de Castro Moura

**Affiliations:** I Universidade Federal de Viçosa. Viçosa, Minas Gerais, Brazil; II Centro Universitário Newton Paiva. Belo Horizonte, Minas Gerais, Brazil; III Universidade Professor Edson Antônio Velano. Alfenas, Minas Gerais. Brazil; IV Universidade Federal de Alfenas. Alfenas, Minas Gerais, Brazil; V Universidade Federal de Minas Gerais. Belo Horizonte, Minas Gerais, Brazil

**Keywords:** Students, Universities, COVID-19, Academic Performance, Mental Health, Estudiantes, Universidades, COVID-19, Rendimiento Académico, Salud Mental

## Abstract

**Objectives:**

to evaluate the prevalence of negative affectivity in university students in the post-COVID-19 pandemic context and its relationship with academic performance and professional outlook.

**Methods:**

a cross-sectional study was conducted with undergraduate students from a public university in Minas Gerais between September 2022 and September 2023. Data were collected using a sociodemographic and psychosocial characterization questionnaire and the Depression, Anxiety, and Stress Scale 21. The relationships between negative affectivity, academic performance, and professional outlook were verified using the Kruskal-Wallis test, with a significance level of 5%.

**Results:**

a total of 585 students participated in the study. A high prevalence of depression, anxiety, and stress was found among university students in the post-COVID-19 context, with a notable severity of anxiety. A negative association was detected between the investigated negative affectivity, academic performance, and professional outlook.

**Conclusions:**

the results indicate an emotional vulnerability in university students, with a relationship between negative affectivity and a decline in academic performance and professional outlook.

## RESUMEN

**Objetivos:**

evaluar la prevalencia de afectividades negativas en estudiantes universitarios en el contexto post-pandemia de COVID-19 y su relación con el rendimiento académico y la perspectiva de futuro profesional.

**Métodos:**

estudio transversal realizado con estudiantes de pregrado de una universidad pública de Minas Gerais entre septiembre de 2022 y septiembre de 2023. Los datos fueron recolectados a partir de un cuestionario de caracterización sociodemográfica y psicosocial y de la *Depression, Anxiety and Stress Scale* 21. Las relaciones entre las afectividades negativas, el rendimiento escolar y la perspectiva de futuro profesional fueron verificadas mediante la prueba de Kruskal-Wallis, con un nivel de significancia del 5%.

**Resultados:**

participaron en el estudio 585 estudiantes. Se verificó una alta prevalencia de depresión, ansiedad y estrés entre los estudiantes universitarios en el contexto post-pandemia de COVID-19, destacándose la ansiedad en un nivel severo. Se detectó una asociación negativa entre las afectividades negativas investigadas, el rendimiento académico y la perspectiva de futuro profesional.

**Conclusiones:**

los resultados indican una vulnerabilidad emocional en los estudiantes universitarios, con la existencia de una relación entre las afectividades negativas y un deterioro del rendimiento académico y de la perspectiva de futuro profesional.

## INTRODUCTION

Negative affectivity is defined as the individual tendency to experience emotional states characterized by aversive conditions such as depression, anxiety, and stress, which can have a detrimental impact on mental health^(1)^. Mental health problems arise when a person is unable to adapt to changes in their life, resulting in a debilitating state of significant emotional suffering^(2)^ These problems represent a major public health issue. Globally, approximately one in eight people suffers from a mental disorder, making these disorders the leading cause of disability, accounting for one in every six years lived with disability^(2)^. People with severe mental health impairments die on average ten to twenty years earlier than the general population^(2)^.

The years 2020 to 2022 were marked by the emergence of the COVID-19 pandemic, which necessitated the adoption of isolation measures to prevent the spread of the virus, leading to changes in various aspects of life. These changes had a significant psychological impact, triggering mental health symptoms in healthy individuals and exacerbating them in those with pre-existing psychiatric conditions^(3)^. A report published by the World Health Organization in June 2022 indicated that depression and anxiety increased by more than 25% in the first year of the pandemic alone^(2)^. Additionally, the report noted that the pandemic itself was a factor contributing to the increased incidence of stress among the population^(2)^.

In the academic context, specifically, the sudden suspension of in-person activities in educational institutions and the adoption of remote learning models to adapt to the new reality led to the aggravation and increased incidence of negative affectivity among university students^(4)^. A study conducted at a Portuguese university found that levels of depression, anxiety, and stress among students were higher during the pandemic compared to the pre-pandemic period^(5)^.

A widely used instrument for tracking negative affectivity, especially among university students^(5)^, is the Depression, Anxiety, and Stress Scale in its abbreviated 21-item version (DASS-21)^(6)^. This scale was developed to assess and distinguish, to the greatest extent, the signs of anxiety and depression, as well as to establish the dimension of stress assessment as a complement to the measurement of affective disorders^(7)^. This tripartite instrument is organized and structured around the symptoms of each disorder: depression assesses the presence of negative affect symptoms (inertia, lack of pleasure and interest, dysphoria, devaluation of life, and despondency); anxiety assesses the excitation of the autonomic nervous system, musculoskeletal effects, situational anxiety, and subjective experiences of anxiety; and stress assesses difficulty in relaxing, nervous excitation, easy disturbance/agitation, irritability/overreaction, and impatience^(7)^.

Mental health problems negatively affect various aspects of life. Specifically, in university students, negative affectivity can lead to a decrease in academic performance^(8)^. It can also interfere with students’ professional future outlook, directly associating with dropping out of school^(5)^. It is estimated that students experiencing negative affectivity are twice as likely to abandon university^(8)^.

The scientific literature presents studies investigating the relationship between depression and academic performance^(9-11)^; however, the relationships between anxiety and stress with this variable are still emerging, especially in a post-crisis context that has significantly worsened mental health problems in this population. The relationship between negative affectivity and professional future outlook is also scarcely described in the literature, particularly in the post-pandemic context. Therefore, it is essential to conduct investigations that evaluate the relationship between the most prevalent negative affectivities among university students, particularly anxiety, stress, and depression, with academic performance and professional future outlook in the post-COVID-19 pandemic context.

## OBJECTIVES

To evaluate the prevalence of negative affectivity in university students in the post-COVID-19 pandemic context and its relationship with academic performance and professional future outlook.

## METHODS

### Ethical Aspects

This study was conducted in accordance with national^(12)^ and international^(13)^ ethical guidelines and was approved by the Research Ethics Committee of the Federal University of Viçosa, whose decision is attached to this submission. Informed consent was obtained from all individuals involved in the study through an online declaration. Students who agreed to participate were instructed to click the “I have read and agree to participate in the study” button. Students who did not agree to participate were instructed to close the browser page.

### Design, Study Location, and Period

This cross-sectional study, reported according to the Strengthening the Reporting of Observational Studies in Epidemiology (STROBE) guidelines^(14)^, was conducted across the three campuses of a federal public university in Minas Gerais from September 2022 to September 2023. The selected university offers 67 undergraduate programs in various fields of knowledge and has approximately 14,000 university students.

### Population and Sample; Inclusion and Exclusion Criteria

The study population consisted of the 14,000 undergraduate students from the three campuses of the university. The sample was obtained by convenience sampling, as all students from the three campuses were invited to participate in the research. Inclusion criteria included being regularly enrolled in any period of the undergraduate courses offered by the university, being 18 years or older, and being available to respond to the data collection instruments online. Students who responded incompletely to the instruments were excluded.

### Study Protocol

The research was disseminated through the university’s official communication channels, institutional emails, and by distributing flyers. Students who agreed to participate in the study signed the informed consent form digitally. They then responded to the data collection instruments online: the sociodemographic and psychosocial characterization questionnaire and the Depression, Anxiety, and Stress Scale 21 (DASS-21)^(15)^.

The characterization questionnaire was used to outline the sociodemographic and psychosocial profiles of the participants. The following variables were considered: gender, age, course of study, psychiatric and psychological follow-ups, clinical diagnosis related to mental health, use of psychotropic medications, whether the student perceived that the COVID-19 pandemic compromised their academic performance and professional future outlook, feelings of being overwhelmed with academic activities, and self-assessment of physical and mental health. Before application, this questionnaire was evaluated by four professionals with experience in mental health and was completed by three university students to verify the comprehension of its items, who judged it adequate.

The DASS-21^(15)^ was used to map the levels of negative affectivity - depression, anxiety, and stress - among the study participants. This self-administered instrument consists of 21 questions divided into three subscales, with seven questions each, assessing symptoms in the past week and offering four response options (0 = did not apply at all; 1 = applied to some degree, or for a short time; 2 = applied to a considerable degree or for a good part of the time; 3 = applied very much or most of the time). The sum of each subscale multiplied by two provides the total score for each construct, indicating the level of depression, anxiety, and stress, which can range from normal, low, moderate, severe, and extremely severe. The cutoff points for depression are: 0-9 (normal); 10-13 (low); 14-20 (moderate); 21-27 (severe); and >27 (extremely severe). For anxiety: normal (0-7); low (8-9); moderate (10-14); severe (15-19); and extremely severe (>20). And for stress: normal (0-14); low (15-18); moderate (19-25); severe (26-33); and extremely severe (>33)^(15)^.

To calculate the score of each construct of the DASS-21, a spreadsheet was created in Microsoft Excel®, version 2021, with the assistance of a statistician, including automatic formulas to generate results for each construct, as well as stratification by levels: normal, low, moderate, severe, and extremely severe. The DASS-21 was translated and validated for the Brazilian version and has adequate psychometric properties, with a total Cronbach’s alpha of 0.96; being 0.93 for the depression subscale, 0.91 for the stress subscale, and 0.86 for the anxiety subscale^(15)^.

### Analysis of Results and Statistics

The collected data were analyzed using the Statistical Package for the Social Sciences® (SPSS), version 23.0. Absolute and relative frequencies were used to describe categorical variables. Continuous variables were described using mean and standard deviation, as well as median and interquartile range, given the absence of normality. To verify the relationship between negative affectivity and the variables of academic performance and professional future outlook, the non-parametric Kruskal-Wallis association test was employed, with a significance level of 5%.

## RESULTS

A total of 618 students agreed to participate in this study, with 33 being excluded for providing incomplete responses to the data collection instruments. The final sample consisted of 585 students. The average age was 23.01 years (standard deviation: 4.45; median: 22; interquartile range: 20-24). The self-assessment of physical and mental health after the pandemic, evaluated on a scale from zero (very poor) to 10 (excellent), showed averages of 6.12 (standard deviation: 2.10; median: 6; interquartile range: 5-8) and 5.08 (standard deviation: 2.06; median: 5; interquartile range: 4-6), respectively. Table 1 presents the other sociodemographic and psychosocial characteristics evaluated.

**Table 1:** Sociodemographic and Psychosocial Characteristics of University Students in the Post-COVID-19 Pandemic Context (n=585), Viçosa, Minas Gerais, Brazil, 2024

Sociodemographic and psychosocial characteristics	n*	%^†^
Gender		
Female	416	71.1
Male	169	28.9
Course of study		
Biological and Health Sciences	275	47.0
Human Sciences	151	25.8
Exact Sciences	127	21.7
Agricultural Sciences	32	5.5
Psychological follow-up		
Does not receive	313	53.5
Already received before the pandemic	112	19.1
Started during/after the pandemic	160	27.4
Psychiatric follow-up		
Does not receive	398	68.0
Already received before the pandemic	93	15.9
Started during/after the pandemic	94	16.1
Clinical diagnosis related to mental health		
Anxiety	236	40.3
Depression	116	19.8
Attention Deficit Hyperactivity Disorder (ADHD)	25	4.3
Sleep Disorder	18	3.1
Bipolar Disorder	15	2.6
Burnout Syndrome	13	2.2
Panic Syndrome	12	2.1
Personality Disorder	8	1.4
Obsessive-Compulsive Disorder (OCD)	5	0.9
Social Phobia	5	0.9
Post-Traumatic Stress Disorder (PTSD)	4	0.7
Eating Disorder / Binge Eating Disorder	4	0.7
Autism Spectrum Disorder	1	0.2
Diagnosed during/after the pandemic	169	28.9
Use of psychotropic medications		
Anxiolytic	178	30.4
Antidepressant	166	28.4
Homeopathic / Herbal / Anthroposophic / Flower	26	4.4
Remedies		
Mood Stabilizer	23	3.9
Antipsychotic	17	2.9
Amphetamines	13	2.2
Non-Benzodiazepine Hypnotic	8	1.4
Anticonvulsant	2	0.3
Muscle Relaxant	1	0.2
Started using psychotropic medications during/after the pandemic	162	27.7
Academic performance after the pandemic		
Unchanged	79	13.5
Improved	67	11.5
Worsened	439	75.0
Professional future outlook after the pandemic		
Unchanged	183	31.3
Improved	98	16.8
Worsened	304	52.0
Overload of Academic Activities after the Pandemic	498	85.1

* *n - Absolute Frequency; ^†^% - Relative Frequency.*

The prevalence of depression was 77.8%, anxiety was 74.4%, and stress was 76.8% among university students in the post-COVID-19 pandemic context. Notably, severe levels of negative affectivity were more prevalent, especially anxiety, with a prevalence of 42.6% (Table 2).

**Table 2:** Prevalence of Depression, Anxiety, and Stress in University Students in the Post-COVID-19 Pandemic Context (n=585), Viçosa, Minas Gerais, Brazil, 2024

Negative Affectivity	Normal n* (%^†^)	Low n* (%^†^)	Moderate n* (%^†^)	Severe n* (%^†^)	Extremely Severe n* (%^†^)	DASS 21^II^	
						Mean (SD^§^)	Median (p25-p75^‡^)
Depression	130 (22.2%)	73 (12.5%)	126 (21.5%)	84 (14.4%)	172 (29.4%)	19.48 (11.65)	20 (10-28)
Anxiety	150 (25.6%)	33 (5.6%)	83 (14.2%)	70 (12.0%)	249 (42.6%)	17.19 (11.80)	16 (6-26)
Stress	136 (23.2%)	59 (10.1%)	101 (17.3%)	143 (24.4%)	145 (25.0%)	24.05 (10.88)	24 (16-33)

* *n - Absolute Frequency; ^†^% - Relative Frequency;^‡^ - Interquartile Range from the 25^th^ to the 75^th^ Percentile;^§^ - Standard Deviation;* ^II^ - *Depression, Anxiety and Stress Scale 21.*

When comparing the negative affectivities evaluated by the DASS-21 with the variables of academic performance and professional future outlook, all associations were statistically significant. The means/medians of depression, anxiety, and stress were higher among students who reported a decline in academic performance and professional future outlook after the COVID-19 pandemic (Table 3).

**Table 3:** Relationship between Negative Affectivities and the Influence of the COVID-19 Pandemic on Academic Performance and Professional Future Outlook, according to the Kruskal-Wallis Test (n=585), Viçosa, Minas Gerais, Brazil, 2024

	Depression			Anxiety			Stress		
	Mean (SD*)	Median (p25-p75^†^)	*p* value^‡^	Mean (SD*)	Median (p25-p75^†^)	*p* value^‡^	Mean (SD*)	Median (p25-p75^†^)	*p* value^‡^
Academic Performance									
Unchanged	14.51 (11.76)	12 (4-24)	<0.001	11.57 (11.44)	8 (2-18)	<0.001	19.9 (11.91)	20 (10-28)	<0.001
Improved	17.04 (11.09)	16 (8-26)		16.75 (11.78)	16 (6-26)		23.64 (11.59)	26 (16-34)	
Worsened	20.74 (11.44)	20 (12-30)		18.27 (11.60)	18 (8-28)		24.98 (10.36)	26 (18-34)	
Professional Future Outlook									
Unchanged	15.58 (11.13)	14 (6-24)	<0.001	14.85 (11.71)	14 (4-24)	<0.001	20.82 (11.06)	20 (12-30)	<0.001
Improved	15.37 (11.22)	14 (6-26)		13.84 (11.13)	12 (4-22)		21.18 (11.37)	24 (12-30)	
Worsened	23.14 (10.90)	24 (16-32)		19.68 (11.53)	18 (10-28)		26.91 (9.81)	28 (20-34)	

* *Standard Deviation;^†^ - Interquartile Range from the 25^th^ to the 75^th^ Percentile;^‡^ - Kruskal-Wallis Test.*

## DISCUSSION

A high prevalence of depression, anxiety, and stress was observed among university students in the post-COVID-19 pandemic context, with severe anxiety being particularly prominent. Furthermore, an association was found between the investigated triad, academic performance, and professional future outlook, with higher means among students who reported that their academic performance and professional future outlook worsened due to the COVID-19 pandemic.

Investigations conducted during the two years of the pandemic helped to understand its negative impact on the mental health of the university population^(4,16-17)^. Continuing studies in the post-pandemic context is important to identify the behavior of negative affectivities among university students (whether they remained the same, worsened, or improved) and the consequences they may have on academic performance and professional future outlook, in order to guide mental health promotion actions for this population.

In the DASS-21 evaluation, severe anxiety was the most prevalent clinical condition among the study participants. Additionally, anxiety was the most reported mental health diagnosis among students, and anxiolytics were the most commonly used class of psychotropic medications. Similarly, a study conducted with Brazilian health science students found a higher prevalence of severe and extreme anxiety among participants compared to depression and stress^(18)^.

Anxiety is a physiological emotional response to a stressor; however, when this feeling becomes excessive to the point of interfering with daily activities, it becomes pathological^(19)^. The academic routine is marked by numerous stressors, such as task overload, personal and family demands, social life requirements, and concerns about the professional future; all these factors make university students more susceptible to developing anxiety disorders if they do not have sufficient resilience to cope with these stressors^(20)^. Anxiety is directly related to the development of other psychosomatic illnesses and is one of the most common causes of disability^(1)^, even leading to economic losses. Therefore, it is essential that institutions pay attention to the prevalence of this disorder among their students and adopt measures to prevent it.

Most participants in this study denied receiving psychological or psychiatric follow-up. These findings are concerning, especially considering the high prevalence of negative affectivity among the participants. Follow-up by qualified professionals is essential for the adequate treatment of students experiencing mental distress^(21)^. Therefore, it is crucial that educational institutions engage in discussions and interactions with the management bodies of the Unified Health System (SUS), which is one of the most important state actions contributing to the population’s well-being, regarding the mental health demands of the academic community. This will ensure the implementation of strategic planning actions and health interventions that address this population, thus guaranteeing the promotion of mental health.

Among the participants who reported having a mental health diagnosis, the number of students diagnosed before the pandemic was higher than those diagnosed during/after the pandemic. Despite the significant negative impact of the pandemic on university students’ mental health^(4)^, studies prior to COVID-19 already indicated a greater susceptibility of the university population to mental illness compared to the non-university population^(17, 22)^.

The greater vulnerability of university students to developing mental health problems can be justified by the adaptations inherent to this period of formation, which commonly coincides with the transition to adulthood, marked by various physical, psychological, and social changes^(23)^. The need to reconcile these changes with academic tasks puts students under great pressure, making them more susceptible to mental illness. Additionally, the high workload reduces their hours of sleep, physical exercise, and leisure activities, all of which are predisposing factors for the development of mental disorders^(23)^.

With COVID-19, new stressors emerged in the academic context, such as the fear of contamination, concerns about delays in course completion, and the need to adapt to the remote learning model, which negatively affected the mental health of the university population^(3)^. Corroborating this evidence, in the present study, participants presented a median self-assessment of mental health after the COVID-19 pandemic. Moreover, most students who reported receiving psychiatric follow-up, psychological follow-up, and using psychotropic medications started doing so during/after the pandemic, highlighting the negative impact of COVID-19 on the mental health of the university population.

Besides the psychological impact, the COVID-19 pandemic also affected the physical health of the university population. With distance learning, students spent more time sitting or lying down, reduced physical activity, increased screen time, adopted unhealthy eating habits, and experienced sleep problems, all contributing to the increased incidence of chronic diseases among this population, such as obesity and depression^(24)^. Consequently, in this study, participants presented a median self-assessment of physical health similar to their mental health after the COVID-19 pandemic.

Additionally, most participants reported feeling more overwhelmed with the academic routine after the pandemic. The academic routine itself imposes a greater burden on university students, who are constantly pressured to achieve academic and professional success, in addition to facing exhaustive study hours and not having enough time for leisure activities^(20)^. The return to in-person activities in educational institutions after the stabilization provided by vaccination was accompanied by great insecurity and fear, requiring measures to avoid virus contamination, which increased the stress load on university students. Therefore, it is essential that, with the resumption of in-person activities, university faculty and administrators pay attention to the feeling of overload among their students, organizing teaching tasks in a balanced way and providing immediate guidance and help to prevent it^(24)^.

In the present study, most participants stated that the pandemic worsened their academic performance. This result corroborates the findings of an investigation conducted at a Brazilian university, in which 49.5% of students reported insufficient academic performance during the COVID-19 pandemic^(25)^. With the adoption of remote learning, several aspects hindered the teaching and learning process, such as stress caused by social distancing, the need to adapt the study environment to the home environment and reconcile the academic routine with household activities, difficulty accessing the internet and adapting to digital resources, and lack of teacher preparation, which interfered with students’ academic performance during the pandemic period^(25)^.

For most participants in this study, the pandemic worsened their professional future outlook. Fear and insecurity about the future due to the pandemic, concerns about delays in course completion due to the suspension of in-person classes, and decreased academic performance may have negatively influenced university students’ professional future outlook. It is important to pay attention to this finding because a poor professional future outlook is directly related to a higher chance of dropping out of school^(26)^.

A negative association was found between the investigated negative affectivities, academic performance, and professional future outlook, with participants who recognized that the pandemic worsened their academic performance and professional future outlook presenting higher levels of depression, anxiety, and stress. Mental disorders in early adulthood lead to the development of cognitive impairments^(27)^, which justifies the association between negative affectivities and worsening academic performance. Moreover, the long-term consequences associated with mental health problems lead to issues in the job market^(28)^, which may justify the negative influence on the professional future outlook of university students.

These findings are concerning, especially considering the high incidence of negative affectivity among university students during the COVID-19 pandemic^(4)^. In this context, with the resumption of in-person activities in the post-pandemic period, it is essential that educational institutions provide psychopedagogical support to students with mental health needs and coordinate their follow-up within the SUS health care network to ensure their academic performance and positive professional future outlook.

For future investigations, it is suggested to use statistical approaches adjusted for confounding factors, allowing a deeper understanding of the relationships between negative affectivity, academic performance, and professional future outlook. Additionally, it is recommended to conduct longitudinal and multicentric studies to observe the relationship between the studied variables over time. It is also important to explore interventions aimed at addressing negative affectivity among university students in the post-COVID-19 pandemic context to contribute to the promotion of mental health in this population.

### Study limitations

A limitation of the present study is the statistical analysis method employed, which did not consider adjustments for confounding factors, limiting itself to only the correlation between outcomes. Including such analysis could provide a more accurate interpretation of the results. Additionally, the convenience sampling and conducting the investigation at a single institution are factors that contributed to restricting the generalizability of the results. Moreover, the fact that data collection was conducted online increased the chances of selection bias, as only students with internet access could participate in the study.

However, it is noteworthy that despite these limitations, this study was conducted with methodological rigor, presented a significant sample size, and used validated instruments for measuring variables, providing reliable results.

### Contributions to the Field of Health

The findings of this study contribute to the advancement of scientific knowledge in the health field by highlighting the most prevalent negative affectivities among university students in the post-COVID-19 pandemic context and their influence on academic performance and professional future outlook. Besides the interference with academic performance and professional future outlook, negative affectivities among university students can lead to maladaptive behaviors such as excessive alcohol consumption, smoking, substance abuse, overeating, risky sexual activities, social media dependency, sleep deprivation, and suicidal ideation^(4)^. In this sense, the findings of this study justify the importance and urgency of adopting actions aimed at promoting the mental health of the university population.

## CONCLUSIONS

The results indicate an emotional vulnerability among university students in the post-COVID-19 pandemic context, with a prevalence of severe anxiety. Additionally, there was a verified relationship between negative affectivities and poorer academic performance and professional future outlook.

## Data Availability

https://doi.org/10.48331/scielodata.ZADXTC
