# Supplementary material for: Negative affectivity in university students and its relationship with academic performance and professional outlook after COVID-19
Source: Rev Bras Enferm. 2024 Aug 30;77(Suppl 1):e20240040. doi: 10.1590/0034-7167-2024-0040 (PMC11368382; doi:10.1590/0034-7167-2024-0040)
Supplement: Translation

# Translation: Afetividades negativas em universitários e relação com desempenho acadêmico e perspectiva profissional após a COVID-19

**DOI:** 10.1590/0034-7167-2024-0040pt

**Authors:** Bruna de Oliveira Alves, Bárbara Guimarães Lourenço, Bianca Bacelar Assis Araújo, Luana Vieira Toledo, Rafael Lopes Chaves, Érika de Cássia Lopes Chaves, Tânia Couto Machado Chianca, Caroline de Castro Moura

**Affiliations:** I Universidade Federal de Viçosa. Viçosa, Minas Gerais, Brasil; II Centro Universitário Newton Paiva. Belo Horizonte, Minas Gerais, Brasil; III Universidade Professor Edson Antônio Velano. Alfenas, Minas Gerais. Brasil; IV Universidade Federal de Alfenas. Alfenas, Minas Gerais, Brasil; V Universidade Federal de Minas Gerais. Belo Horizonte, Minas Gerais, Brasil

**Keywords:** Estudantes, Universidades, COVID-19, Desempenho Acadêmico, Saúde Mental

## Abstract

**Objetivos:**

avaliar a prevalência de afetividades negativas em estudantes universitários no contexto pós-pandemia da COVID-19 e sua relação com o desempenho acadêmico e a perspectiva de futuro profissional.

**Métodos:**

estudo transversal realizado com estudantes de graduação de uma universidade pública mineira entre setembro de 2022 e setembro de 2023. Os dados foram coletados a partir do questionário de caracterização sociodemográfica e psicossocial e da *Depression, Anxiety and Stress Scale* 21. As relações entre as afetividades negativas, o rendimento escolar e a perspectiva de futuro profissional foram verificadas por meio do teste de Kruskal-Wallis, com 5% de significância.

**Resultados:**

participaram do estudo 585 estudantes. Verificou-se alta prevalência de depressão, ansiedade e estresse entre os estudantes universitários no contexto pós-pandemia da COVID-19, com destaque para a ansiedade em nível severo. Foi detectada associação negativa entre as afetividades negativas investigadas, o desempenho acadêmico e a perspectiva de futuro profissional.

**Conclusões:**

os resultados apontam para uma vulnerabilidade emocional dos estudantes universitários, com existência de relação entre afetividades negativas e piora do desempenho acadêmico e perspectiva de futuro profissional.

## INTRODUÇÃO

A afetividade negativa é definida como a tendência individual de vivenciar experiências emocionais caracterizadas por estados aversivos como, por exemplo, depressão, ansiedade e estresse, os quais podem exercer um papel prejudicial à saúde mental das pessoas^(1)^. Os problemas de saúde mental surgem quando a pessoa não consegue se adaptar às mudanças que ocorrem em sua vida, gerando um estado debilitante de grande sofrimento emocional^(2)^. Eles representam um importante problema de saúde pública. Em todo o mundo, aproximadamente uma em cada oito pessoas sofre de algum transtorno mental, e eles são a principal causa de incapacidade, causando um em cada seis anos vividos com incapacidade^(2)^. Pessoas com condições graves de comprometimento psíquico morrem, em média, dez a vinte anos mais cedo do que a população em geral^(2)^.

Os anos de 2020 a 2022 foram marcados pela emergência da pandemia da COVID-19, que exigiu a adoção de medidas de isolamento com o objetivo de impedir a propagação do vírus, o que gerou mudanças em diversos aspectos da vida. Essas mudanças trouxeram um significativo impacto psicológico, desencadeando sintomas mentais em pessoas saudáveis e intensificando-os em pessoas com condições psiquiátricas pré-existentes^(3)^. Em relatório publicado pela Organização Mundial da Saúde em junho de 2022, a depressão e a ansiedade aumentaram mais de 25% apenas no primeiro ano da pandemia^(2)^. Ainda de acordo com o relatório, a própria pandemia foi um fator que contribuiu para o aumento da incidência de estresse entre a população^(2)^.

No contexto acadêmico, especificamente, as implicações relacionadas à suspensão repentina das atividades presenciais nas instituições de ensino e à adoção do modelo remoto em adequação à nova realidade levaram ao agravamento e ao aumento da incidência das afetividades negativas entre os estudantes universitários^(4)^. Uma investigação realizada em uma universidade portuguesa verificou que os níveis de depressão, ansiedade e estresse entre os estudantes foram maiores durante a pandemia do que no período anterior^(5)^.

Um instrumento amplamente utilizado no rastreamento das afetividades negativas, especialmente em estudantes universitários^(5)^, é a *Depression, Anxiety and Stress Scale,* em sua versão abreviada de 21 itens (DASS-21)^(6)^. Essa escala foi desenvolvida com o objetivo de aferir e distinguir, ao extremo, os sinais de ansiedade e depressão, bem como estabelecer a dimensão da avaliação do estresse como complemento da medida dos transtornos afetivos^(7)^. Esse instrumento tripartido se organiza e se estrutura nos sintomas de cada transtorno, em que a depressão avalia a presença de sintomas de afeto negativo (inércia, falta de prazer e interesse, disforia, desvalorização da vida e desânimo); a ansiedade avalia a excitação do sistema nervoso autônomo, os efeitos musculoesqueléticos, a ansiedade situacional e as experiências subjetivas de ansiedade; e o estresse avalia a dificuldade em relaxar, a excitação nervosa, a perturbação/agitação fácil, a irritabilidade/reação exagerada e a impaciência^(7)^.

Os problemas de saúde mental afetam negativamente vários aspectos da vida. Nos estudantes universitários, especificamente, as afetividades negativas podem ocasionar diminuição do desempenho acadêmico^(8)^. Podem interferir, ainda, na perspectiva de futuro profissional dos estudantes, associando-se diretamente ao abandono dos estudos^(5)^. Estima-se que estudantes que vivenciam afetividades negativas têm duas vezes mais chances de abandonar a universidade^(8)^.

A literatura científica apresenta estudos que investigam a relação entre a depressão e o desempenho acadêmico^(9-11)^; entretanto, as relações entre a ansiedade e o estresse com essa variável são incipientes, ainda mais em um contexto pós-crise, que agravou substancialmente os problemas de saúde mental nesse público. A relação entre afetividades negativas e perspectiva de futuro profissional também é pouco descrita na literatura, principalmente no contexto pós-pandêmico. Sendo assim, destaca-se a necessidade de investigações que avaliem a relação entre as afetividades negativas mais prevalentes entre os estudantes universitários, destacando-se a ansiedade, o estresse e a depressão, com o desempenho acadêmico e a perspectiva de futuro profissional no contexto pós-pandemia da COVID-19.

## OBJETIVOS

Avaliar a prevalência de afetividades negativas em estudantes universitários no contexto pós-pandemia da COVID-19 e sua relação com o desempenho acadêmico e a perspectiva de futuro profissional.

## MÉTODOS

### Aspectos éticos

Este estudo foi conduzido de acordo com as diretrizes de ética nacionais^(12)^ e internacionais^(13)^ e aprovado pelo Comitê de Ética em Pesquisa da Universidade Federal de Viçosa, cujo parecer está anexado à presente submissão. O consentimento livre e esclarecido foi obtido de todos os indivíduos envolvidos no estudo por meio de declaração *online.* Os estudantes que concordaram em participar foram orientados a clicarem no botão “li e concordo em participar da pesquisa”. Os estudantes que não concordaram com a participação foram orientados a fechar a página no navegador.

### Desenho, local do estudo e período

Estudo transversal, reportado segundo o *Strengthening the Reporting of Observational Studies in Epidemiology* (STROBE)^(14)^ e realizado nos três campi de uma universidade pública federal mineira, no período de setembro de 2022 a setembro de 2023.

A universidade escolhida para realização do estudo oferece 67 cursos de graduação em diferentes áreas do conhecimento e possui cerca de 14.000 estudantes universitários.

### População e amostra; critérios de inclusão e exclusão

A população do presente estudo foi composta pelos 14.000 estudantes de graduação dos três campi da universidade. A amostra foi obtida por conveniência, à medida em que todos os estudantes dos três campi foram convidados a colaborar com a pesquisa.

Foram adotados como critérios de inclusão estar regulamente matriculado em qualquer período dos cursos de graduação oferecidos pela universidade, ter 18 anos ou mais e ter disponibilidade para responder aos instrumentos de coleta de dados de forma *online.* Foram excluídos os estudantes que responderam aos instrumentos de forma incompleta.

### Protocolo do estudo

A pesquisa foi divulgada nos canais de comunicação oficial da universidade, nos e-mails institucionais e por meio de entrega de panfletos. Os estudantes que concordaram em participar da pesquisa assinaram o termo de consentimento livre e esclarecido em formato digital. Em seguida, responderam aos instrumentos de coleta de dados, de forma online: o questionário de caracterização sociodemográfica e psicossocial e a *Depression, Anxiety and Stress Scale* 21 (DASS-21)^(15)^.

O questionário de caracterização foi utilizado para traçar o perfil sociodemográfico e psicossocial dos participantes. Para isso, foram consideradas as seguintes variáveis: sexo; idade; curso; acompanhamentos psiquiátrico e psicológico; diagnóstico clínico relacionado à saúde mental; uso de medicamentos psicotrópicos; se o estudante percebeu que a pandemia da COVID-19 comprometeu seu desempenho acadêmico e sua perspectiva de futuro profissional; sentimento de sobrecarga com as atividades acadêmicas; e autoavaliação das saúdes física e mental. Antes de ser aplicado, esse questionário passou por avaliação de quatro profissionais com experiência na área de saúde mental e foi respondido por três estudantes universitários com o objetivo de verificar a compreensão de seus itens, que o julgaram adequado.

A DASS-21^(15)^ foi utilizada para mapear os níveis das afetividades negativas - depressão, ansiedade e estresse - entre os participantes do estudo. Esse instrumento autoaplicável possui 21 questões divididas em três subescalas, com sete perguntas cada, que avaliam sintomas na última semana e possuem quatro opções de resposta (0 = não se aplicou de maneira alguma; 1 = aplicou-se em algum grau, ou por pouco tempo; 2 = aplicou-se em um grau considerável ou por uma boa parte do tempo; 3 = aplicou-se muito ou na maioria do tempo). O somatório de cada subescala multiplicado por dois fornece o escore total de cada construto, indicando o nível de depressão, ansiedade e estresse, que pode variar entre normal, baixo, moderado, grave e severo. Os pontos de corte para depressão são: 0-9 (normal); 10-13 (baixo); 14-20 (moderado); 21-27 (grave); e >27 (extremamente grave). Para ansiedade: normal (0-7); baixa (8-9); moderada (10-14); grave (15-19); e extremamente grave (>20). E para o estresse: normal (0-14); baixo (15-18); moderado (19-25); grave (26-33); e extremamente grave (>33)^(15)^.

Para calcular o escore de cada construto da DASS-21, foi criada uma planilha no *software Microsoft Excel*®*,* versão 2021, com auxílio de um estatístico, com fórmulas automáticas para gerar resultados de cada construto, e também a estratificação por níveis normal, baixo, moderado, grave e severo. A DASS-21 foi traduzida e validada para a versão brasileira e possui adequadas propriedades psicométricas, com alfa de Cronbach total de 0,96; sendo 0,93 para a subescala de depressão, 0,91 para a subescala de estresse e 0,86 para a subescala de ansiedade^(15)^.

### Análise dos resultados e estatística

Os dados coletados foram analisados por meio do *software* estatístico *Statistical Package for the Social Sciences*®*,* versão 23.0. As frequências absoluta e relativa foram utilizadas para a descrição das variáveis categóricas. As variáveis contínuas foram descritas por meio de média e desvio padrão, e também por mediana e intervalo interquartílico, dada ausência de normalidade. A fim de verificar a relação entre as afetividades negativas e as variáveis rendimento escolar e perspectiva de futuro profissional, empregou-se o teste de associação não paramétrico de Kruskall Wallis, à 5% de significância.

## RESULTADOS

Aceitaram participar deste estudo 618 estudantes, sendo 33 excluídos por responderem aos instrumentos de coleta de dados de forma incompleta. A amostra final foi constituída por 585 estudantes. A média de idade foi de 23,01 anos (desvio padrão: 4,45; mediana: 22; distância interquartílica: 20-24). A autopercepção das saúdes física e mental após a pandemia, avaliada entre zero (muito ruim) e 10 (excelente), apresentou médias de 6,12 (desvio padrão: 2,10; mediana: 6; distância interquartílica: 5-8) e 5,08 (desvio padrão: 2,06; mediana: 5; distância interquartílica: 4-6), respectivamente. A Tabela 1 apresenta as demais características sociodemográficas e psicossociais avaliadas.

**Tabela 1:** Caracterização sociodemográfica e psicossocial dos estudantes universitários no contexto pós pandemia da COVID-19 (n=585), Viçosa, Minas Gerais, Brasil, 2024

Características sociodemográficas e psicossociais	n*	%^†^
Sexo		
Feminino	416	71,1
Masculino	169	28,9
Curso		
Ciências biológicas e da saúde	275	47,0
Ciências humanas	151	25,8
Ciências exatas	127	21,7
Ciências agrárias	32	5,5
Acompanhamento psicológico		
Não faz	313	53,5
Já fazia antes da pandemia	112	19,1
Começou a fazer durante/após a pandemia	160	27,4
Acompanhamento psiquiátrico		
Não faz	398	68,0
Já fazia antes da pandemia	93	15,9
Começou a fazer durante/após a pandemia	94	16,1
Diagnóstico clínico relacionado à saúde mental		
Ansiedade	236	40,3
Depressão	116	19,8
Transtorno do déficit de atenção com hiperatividade	25	4,3
Transtorno do sono	18	3,1
Transtorno bipolar	15	2,6
Síndrome de burnout	13	2,2
Síndrome do pânico	12	2,1
Transtorno de personalidade	8	1,4
Transtorno obsessivo-compulsivo	5	0,9
Fobia social	5	0,9
Transtorno de estresse pós-traumático	4	0,7
Transtorno alimentar / compulsão alimentar	4	0,7
Transtorno do espectro autista	1	0,2
Diagnóstico estabelecido durante/após a pandemia	169	28,9
Utilização de medicamentos psicotrópicos		
Ansiolítico	178	30,4
Antidepressivo	166	28,4
Homeopático / fitoterápico / antroposófico / florais	26	4,4
Estabilizador do humor	23	3,9
Antipsicótico	17	2,9
Anfetaminas	13	2,2
Hipnótico não benzodiazepínico	8	1,4
Anticonvulsivante	2	0,3
Relaxante muscular	1	0,2
Início do uso de medicamentos psicotrópicos durante/após a pandemia	162	27,7
Desempenho acadêmico após pandemia		
Não alterou	79	13,5
Melhorou	67	11,5
Piorou	439	75,0
Perspectiva de futuro profissional após pandemia		
Não alterou	183	31,3
Melhorou	98	16,8
Piorou	304	52,0
Sobrecarga das atividades acadêmicas após pandemia	498	85,1

* *n - Frequência absoluta; ^†^% - Frequência relativa.*

A prevalência de depressão foi de 77,8%, de ansiedade, 74,4% e de estresse, 76,8% entre os estudantes universitários no contexto pós pandemia da COVID-19. Destaca-se o nível severo, em que as afetividades negativas foram mais prevalentes, em especial a ansiedade (prevalência de 42,6%) (tabela 2).

**Tabela 2:** Prevalência de depressão, ansiedade e estresse em estudantes universitários no contexto pós-pandemia da COVID-19 (n=585), Viçosa, Minas Gerais, Brasil, 2024

Afetividade negativa	Normal n* (%^†^)	Baixo n* (%^†^)	Moderado n* (%^†^)	Grave n* (%^†^)	Severo n* (%^†^)	DASS 21^II^	
						Média (DP^§^)	Mediana (p25-p75^‡^)
Depressão	130 (22,2%)	73 (12,5%)	126 (21,5%)	84 (14,4%)	172 (29,4%)	19,48 (11,65)	20 (10-28)
Ansiedade	150 (25,6%)	33 (5,6%)	83 (14,2%)	70 (12,0%)	249 (42,6%)	17,19 (11,80)	16 (6-26)
Estresse	136 (23,2%)	59 (10,1%)	101 (17,3%)	143 (24,4%)	145 (25,0%)	24,05 (10,88)	24 (16-33)

* *n - Frequência absoluta; %^†^ - Frequência relativa;^‡^ - Distância interquartílica do percentil 25 ao percentil 75;^§^ - Desvio padrão;* ^II^ - *Depression, Anxiety and Stress Scale 21.*

Na comparação entre as afetividades negativas avaliadas pela DASS 21 com as variáveis desempenho acadêmico e perspectiva de futuro profissional, todas as associações foram estatisticamente significativas. As médias/medianas de depressão, ansiedade e estresse foram maiores entre os estudantes que apontaram piora do desempenho acadêmico e da perspectiva de futuro profissional após a pandemia da COVID-19 (Tabela 3).

**Tabela 3:** Relação entre afetividades negativas e a influência da pandemia da COVID-19 no desempenho acadêmico e na perspectiva de futuro profissional, segundo teste de Kruskall Wallis (n=585), Viçosa, Minas Gerais, Brasil, 2024

	Depressão			Ansiedade			Estresse		
	Média (DP*)	Mediana (p25-p75^†^)	Valor de *p* ^‡^	Média (DP*)	Mediana (p25-p75^†^)	Valor de *p* ^‡^	Média (DP*)	Mediana (p25-p75^†^)	Valor de *p* ^‡^
Desempenho acadêmico									
Não alterou	14,51 (11,76)	12 (4-24)	<0,001	11,57 (11,44)	8 (2-18)	<0,001	19,9 (11,91)	20 (10-28)	<0,001
Melhorou	17,04 (11,09)	16 (8-26)		16,75 (11,78)	16 (6-26)		23,64 (11,59)	26 (16-34)	
Piorou	20,74 (11,44)	20 (12-30)		18,27 (11,60)	18 (8-28)		24,98 (10,36)	26 (18-34)	
Perspectiva de futuro profissional									
Não alterou	15,58 (11,13)	14 (6-24)	<0,001	14,85 (11,71)	14 (4-24)	<0,001	20,82 (11,06)	20 (12-30)	<0,001
Melhorou	15,37 (11,22)	14 (6-26)		13,84 (11,13)	12 (4-22)		21,18 (11,37)	24 (12-30)	
Piorou	23,14 (10,90)	24 (16-32)		19,68 (11,53)	18 (10-28)		26,91 (9,81)	28 (20-34)	

* *- Desvio padrão;^†^ - Distância interquartílica do percentil 25 ao percentil 75;^‡^ - Teste de Kruskall Wallis.*

## DISCUSSÃO

Evidenciou-se alta prevalência de depressão, ansiedade e estresse entre os estudantes universitários no contexto pós-pandemia da COVID-19, com destaque para a ansiedade em nível severo. Ademais, foi verificada associação entre a tríade investigada, o desempenho acadêmico e a perspectiva de futuro profissional, de forma que as médias foram maiores entre os estudantes que afirmaram que seu desempenho acadêmico e sua perspectiva de futuro profissional pioraram com a pandemia da COVID-19.

As investigações realizadas durante os dois anos de pandemia ajudaram a entender seu impacto negativo sobre a saúde mental da população universitária^(4,16-17)^. E, a continuidade dos estudos no contexto pós-pandêmico é importante por possibilitar a identificação do comportamento das afetividades negativas entre os estudantes universitários (mantiveram-se da mesma forma, pioraram ou melhoraram) e as consequências que podem trazer para o desempenho acadêmico e para a perspectiva de futuro profissional, a fim de direcionar as ações de promoção da saúde mental nessa população.

Na avaliação da DASS-21, a ansiedade em nível severo foi a condição clínica mais prevalente entre os participantes do estudo. Além disso, a ansiedade foi o diagnóstico relacionado à saúde mental que mais estudantes referiram possuir, e os ansiolíticos foram a classe de medicamentos psicotrópicos mais utilizada por eles. De forma semelhante, estudo realizado com estudantes brasileiros da área da saúde verificou maior prevalência de ansiedade nos níveis grave e severo entre os participantes em relação à depressão e ao estresse^(18)^.

A ansiedade é uma resposta emocional fisiológica a um estressor; no entanto, quando esse sentimento é excessivo, a ponto de interferir na execução das atividades de vida diária, torna-se patológica^(19)^. A rotina acadêmica é marcada por inúmeros estressores, como sobrecarga de tarefas, cobranças pessoais e familiares, exigências da vida social e preocupação com o futuro profissional; todos esses fatores tornam os estudantes universitários mais suscetíveis ao desenvolvimento de transtornos de ansiedade, caso não apresentem resiliência suficiente para lidar com esses estressores^(20)^. A ansiedade está diretamente relacionada com o desenvolvimento de outras doenças psicossomáticas e é uma das causas mais comuns de incapacidade^(1)^, levando até mesmo a perdas econômicas. Nesse sentido, é fundamental que as instituições atentem-se para a prevalência desse transtorno entre seus discentes e adotem medidas para preveni-lo.

A maioria dos participantes do presente estudo negou acompanhamento psicológico ou psiquiátrico. Esses achados são preocupantes, sobretudo ao considerar a elevada prevalência de afetividades negativas entre os participantes. O acompanhamento por profissionais qualificados é fundamental para o adequado tratamento de estudantes em sofrimento mental^(21)^. Torna-se essencial, portanto, que as instituições de ensino realizem discussões e interlocuções com as instâncias gestoras do Sistema Único de Saúde (SUS), que compreende uma das mais importantes ações de Estado que contribui para o bem-estar da população, sobre as demandas de saúde mental da comunidade acadêmica, para que ações de planejamentos estratégicos e intervenções em saúde que contemplem este público sejam implementadas, a fim de garantir a promoção da saúde mental.

Entre os participantes que afirmaram possuir algum diagnóstico relacionado à saúde mental, o número de estudantes diagnosticados antes da pandemia foi maior do que aqueles diagnosticados durante/após a pandemia. Apesar do impacto negativo significativo da pandemia sobre a saúde mental dos estudantes universitários^(4)^, antes da COVΊD-19, estudos já apontavam para uma maior suscetibilidade da população universitária ao adoecimento mental em relação à população não universitária^(17,22)^.

A maior vulnerabilidade dos estudantes universitários ao desenvolvimento de problemas de saúde mental pode ser justificada pelas adaptações inerentes a esse período de formação, que, comumente, coincide com a fase de transição para a vida adulta, marcada por diversas mudanças físicas, psicológicas e sociais^(23)^. A necessidade de conciliar essas mudanças com as tarefas acadêmicas coloca os estudantes sob grande pressão, tornando-os mais suscetíveis ao adoecimento mental. Além disso, a elevada carga de tarefas faz com que eles diminuam suas horas de sono, de prática de exercícios físicos e de atividades de lazer, fatores estes predisponentes para o desenvolvimento de transtornos mentais^(23)^.

Com a COVID-19, novos estressores emergiram no contexto acadêmico, como o medo da contaminação, a preocupação em relação ao atraso na conclusão do curso e a necessidade de adaptação ao modelo de ensino remoto, o que afetou negativamente a saúde mental da população universitária^(3)^. Corroborando essa evidência, no presente estudo, os participantes apresentaram uma autoavaliação mediana da saúde mental após a pandemia da COVID-19. Ademais, a maioria dos estudantes que afirmaram fazer acompanhamento psiquiátrico, acompanhamento psicológico e uso de medicamentos psicotrópicos, iniciou-os durante/após a pandemia, evidenciando o impacto negativo da COVID-19 sobre a saúde mental da população universitária.

Além do impacto psicológico, a pandemia da COVID-19 afetou também a saúde física da população universitária. Com o ensino a distância, os estudantes passaram a ficar mais tempo sentados ou deitados, reduziram a prática de atividades físicas, aumentaram o tempo de tela, adotaram hábitos alimentares não saudáveis e apresentaram problemas no sono, o que contribuiu para o aumento da incidência de doenças crônicas entre essa população, tais como obesidade e depressão^(23)^. Com efeito, no presente estudo, os participantes apresentaram, assim como na saúde mental, uma autoavaliação mediana da saúde física após a pandemia da COVID-19.

Ainda, a maioria dos participantes alegou se sentir mais sobrecarregada com a rotina acadêmica após a pandemia. A própria rotina acadêmica impõe uma maior sobrecarga sobre os estudantes universitários, que são constantemente pressionados a alcançar o sucesso acadêmico e profissional, além de enfrentarem exaustivas horas de estudo e não terem tempo suficiente para realizar atividades de lazer^(20)^. O retorno das atividades presenciais nas instituições de ensino após a estabilização proporcionada pela vacinação foi acompanhado por grande insegurança e medo, e exigiu a adoção de medidas para evitar a contaminação pelo vírus, levando a uma maior carga de estresse sobre os estudantes universitários. Diante disso, é imprescindível que, com a retomada das atividades presenciais, os docentes e os administradores universitários atentem-se ao sentimento de sobrecarga entre seus alunos, organizando as tarefas de ensino de maneira equilibrada e fornecendo orientação e ajuda imediata, a fim de preveni-lo^(24)^.

No presente estudo, a maioria dos participantes afirmou que a pandemia piorou seu desempenho acadêmico. Esse resultado corrobora os achados de uma investigação realizada em uma universidade brasileira, em que 49,5% dos discentes referiram um desempenho acadêmico insuficiente durante a pandemia da COVID-19^(25)^. Com a adoção do ensino remoto, alguns aspectos dificultaram o processo de ensino e aprendizagem, como o estresse causado pelo distanciamento social, a necessidade de adaptar o ambiente de estudos ao ambiente domiciliar e conciliar a rotina acadêmica com as atividades domésticas, a dificuldade de acesso à internet e de adaptação aos recursos digitais e a falta de preparo dos professores, o que interferiu no desempenho acadêmico dos estudantes durante o período pandêmico^(25)^.

Para a maioria dos participantes do presente estudo, a pandemia piorou sua perspectiva de futuro profissional. O medo e a insegurança em relação ao futuro em virtude da pandemia, a preocupação relativa ao atraso na conclusão do curso devido à suspensão das aulas presenciais e a diminuição do desempenho acadêmico podem ter influenciado negativamente a perspectiva de futuro profissional dos estudantes universitários. É importante atentar-se para esse achado, pois uma perspectiva de futuro profissional ruim está diretamente relacionada a uma maior chance de abandono dos estudos^(26)^.

Foi verificada associação negativa entre as afetividades negativas investigadas, desempenho acadêmico e perspectiva de futuro profissional, sendo que os participantes que reconheceram que a pandemia piorou seu desempenho acadêmico e sua perspectiva de futuro profissional apresentaram níveis mais elevados de depressão, ansiedade e estresse. Os transtornos mentais no início da vida adulta levam ao desenvolvimento de deficiências cognitivas^(27)^, o que justifica a associação entre afetividades negativas e piora do desempenho acadêmico. Ademais, as consequências associadas aos problemas de saúde mental repercutem a longo prazo, levando a problemas no mercado de trabalho^(28)^, o que pode justificar a influência negativa na perspectiva de futuro profissional dos estudantes universitários.

Esses achados são preocupantes, sobretudo ao considerar a elevada incidência de afetividades negativas entre os estudantes universitários durante a pandemia da COVID-19^(4)^. Nesse sentido, com a retomada das atividades presenciais no contexto pós-pandêmico, torna-se fundamental que as instituições de ensino prestem apoio psicopedagógico aos seus discentes com alguma demanda de saúde mental e articulem o seu acompanhamento na rede de atenção à saúde do SUS, a fim de garantir o desempenho acadêmico e a perspectiva profissional futura positiva dos mesmos.

Para investigações futuras, sugere-se abordagens estatísticas que sejam ajustadas por fatores de confusão, permitindo uma compreensão mais profunda das relações entre afetividades negativas, desempenho acadêmico e perspectiva de futuro profissional. Além disso, propõe-se também a realização de estudos longitudinais e multicêntricos, a fim de observar a relação entre as variáveis estudadas ao longo do tempo. Destaca-se, ainda, a necessidade de explorar intervenções que tenham como objetivo tratar as afetividades negativas entre estudantes universitários no contexto pós-pandemia da COVID-19, a fim de contribuir com a promoção da saúde mental nesta população.

### Limitações do estudo

Como limitação do presente estudo, evidencia-se o método de análise estatística empregada, que não considerou o ajuste por fatores de confusão, limitando-se apenas à correlação entre os desfechos. A inclusão dessa análise poderia proporcionar uma interpretação mais precisa dos resultados. Destaca-se, ainda, a seleção da amostra por conveniência e a realização da investigação em uma única instituição, fatores que contribuíram para restringir a generalização dos resultados. Ademais, o fato de a coleta dos dados ter sido realizada de forma online aumentou as chances de ocorrência de viés de seleção, uma vez que apenas estudantes com acesso à internet puderam participar do estudo.

Entretanto, destaca-se que, apesar das limitações, este estudo foi conduzido com rigor metodológico, apresentou uma amostra expressiva e utilizou instrumentos validados para a mensuração das variáveis, fornecendo resultados seguros.

### Contribuições para a área da saúde

Os achados do presente estudo contribuem com o avanço do conhecimento científico na área da saúde por evidenciar as afetividades negativas mais prevalentes entre os estudantes universitários no contexto pós-pandemia da COVID-19, e a influência dessas sobre o desempenho acadêmico e a perspectiva de futuro profissional. Além das interferências sobre o desempenho acadêmico e a perspectiva de futuro profissional, as afetividades negativas entre estudantes universitários podem levar à adoção de comportamentos desajustados, como consumo excessivo de álcool; tabagismo; abuso de substâncias; alimentação excessiva; atividades sexuais de risco; dependência de mídias sociais; privação de sono; e ideação suicida^(4)^. Nesse sentido, os achados do presente estudo contribuem para justificar a importância e emergência da adoção de ações voltadas para a promoção da saúde mental da população universitária.

## CONCLUSÕES

Os resultados encontrados apontam para uma vulnerabilidade emocional dos estudantes universitários no contexto pós-pandemia da COVID-19, com prevalência de ansiedade em nível severo. Além disso, foi verificada a existência de relação entre afetividades negativas com um pior desempenho acadêmico e uma pior perspectiva de futuro profissional.

## Data Availability

https://doi.org/10.48331/scielodata.ZADXTC
